# Immediate recall of short stories depends on educational level

**DOI:** 10.1590/S1980-57642009DN20400014

**Published:** 2008

**Authors:** Ricardo Nitrini

**Affiliations:** Behavioral and Cognitive Neurology Unit, Department of Neurology and Cognitive Disorders Reference Center (CEREDIC). Hospital das Clínicas of the University of São Paulo School of Medicine.

**Keywords:** memory, Alzheimer’s disease, mild cognitive impairment, logical memory, education, neuropsychological tests

## Abstract

Memory complaints are frequent in the elderly but the confirmation of memory decline is challenging. Tests employing the recall of paragraphs or short stories have been proposed for the diagnosis of Alzheimer’s disease and amnestic mild cognitive impairment.

**Objectives:**

To evaluate the influence of educational level on immediate recall of short stories.

**Methods:**

A sample of 363 individuals (214 women; median age of 50; median years of schooling of 6; 23 illiterates) without evident physical or mental illnesses were evaluated with simple neuropsychological tests, including the recall of short stories immediately after listening to them read aloud by the examiner.

**Results:**

Age showed an inverse correlation whereas years of schooling showed a direct correlation with the scores on the immediate recall of short stories. As age and years of schooling were inversely correlated, logistic regression was employed, which showed that only years of schooling had an influence on the performance in the test.

**Conclusions:**

In populations with heterogeneous educational background, the recall of short stories cannot be recommended for the diagnosis of memory impairment. It is possible that tests with larger encoding phases are more appropriate for these populations. From a broader perspective, information released by radio or TV, as well as information disseminated orally in public settings such as hospitals, stations or airports may be less well retained by low educated individuals, especially when the information is presented only once.

## Abstract (pt)

Queixas de problemas com a memória são freqüentes em idosos, mas a confirmação de declínio da memória não é simples. Testes que utilizam a recordação de parágrafos ou estórias curtas têm sido propostos para o diagnóstico de doença de Alzheimer e de comprometimento cognitivo leve amnéstico.

**Objetivos:**

Avaliar a influência do nível educacional sobre a recordação imediata de estórias curtas.

**Métodos:**

363 indíviduos (214 mulheres; idade mediana de 50; escolaridade mediana de 6; 23 analfabetos) sem doenças físicas ou mentais evidentes foram avaliados com testes neuropsicológicos simples que incluíram a recordação de estórias simples imediatamente depois da leitura em voz alta pelo examinador.

**Resultados:**

Idade correlacionou-se inversamente enquanto anos de escolaridade correlacionou-se diretamente com os escores na recordação imediata das estórias. Como idade e anos de escolaridade correlacionaram-se inversamente, foi empregada regressão logística que demonstrou que apenas a escolaridade influenciou o desempenho no teste.

**Conclusões:**

Em populações com nível educacional heterogêneo, a recordação de estórias curtas não deve ser recomendada para o diagnóstico de comprometimento da memória. É possível que testes com fases de codificação mais prolongadas ou repetidas sejam mais apropriados para populações deste tipo. A partir de perspectiva mais ampla, informações divulgadas por rádio ou televisão, bem como avisos oralmente apresentados em espaços públicos como hospitais, estações ou aeroportos podem ser menos lembrados por indivíduos de baixa escolaridade, especialmente quando a informação for apresentada uma única vez.

Memory complaints are frequent in the elderly but the confirmation of memory decline is relatively complex, yet very significant in the clinical practice.^1^ Both the DSM-IV and ICD-10 require that memory impairment is confirmed for the diagnosis of dementia.^2,3^ When memory decline is still mild, as in amnestic mild cognitive impairment (aMCI), the diagnosis of memory impairment is based on the presence of self memory complaint, preferentially corroborated by an informant and confirmed by a cognitive test.^4^ Usually, delayed recall tests are considered the best for the diagnosis of the type of memory failure that occurs in AD^5^ and aMCI.^4^ Among test tasks, those depending on the recall of a short story or of a paragraph have been considered well suited for such diagnosis.^4,6-9^

The logical memory test of the Weschler Memory Scale (WMS)^6^ has been recommended for the diagnosis of aMCI.^4,10^ In the logical memory test, the participant is asked to pay attention to a short story that is read aloud by the examiner. Immediately after, the participant is requested to recall as many items as possible, where score on form I of the test is the number of recalled items. After an interval of 30 minutes, during which other cognitive tests are given, the participant is requested for a second time to recall the story, and the score recorded on form II is again the number of recalled items. The test is composed of two short stories, with 25 items each.^6^

Although the stories in the logical memory test of the WMS are very simple, recall may be influenced by educational level as is usual for many other neuropsychological tests. Our aim in this study was to evaluate the impact of educational level on the immediate recall of short stories.

## Methods

### Participants

In 1989 and 1990, relatives or friends of patients of the outpatient clinics of the Hospital das Clínicas da Faculdade de Medicina da Universidade de São Paulo, a university-affiliated hospital, were evaluated using simple neuropsychological tests. Before inclusion, each subject was informed of the non-compulsory participation in the study and had to answer to two questions: “Is your healthy good?”; “Is your capacity for working and dealing with your activities good ?” , with answers being ranked as “yes” (0), “almost good” (0.5) or “no” (1). The same questions were put to an informant, using the same ranking. Therefore, the total score ranged from 0 to 4, and in order to be included the subject had to score no more than one.

### Procedures

Demographic data and information on the level of schooling attained by the participants were gathered. The evaluations were performed by residents and post-graduate students of the Department of Neurology employing the following tests: Mini-mental state examination (MMSE),^11^ verbal fluency (animals in one minute), trail-making A,^12^ Luria’s fist-palm-edge test,^13^ and a modified version of form I of the logical memory test (M-LMI).

The M-LMI was composed of two stories with 23 items each. Before reading the story the examiner called the attention of the subject by saying: “I am going to read a short story to you. Listen to it carefully because when I finish I want you to tell me everything. Are you ready?” The first story was taken from the WMS and describes stealing of money from a woman. The second story was created from regular TV or radio news and was as follows: Thursday/ dawn, /a bus/ traveling from Rio/ to São Paulo/ crashed into/ a truck/ loaded with cement./ Thirteen passengers/ were wounded/ two/ of them severely/. The truck’s driver/ died/ at the site of the accident./ According to the police commander/ the bus/ was driving at excessive speed/ and there was fog on the road./ This is the third accident/ of great magnitude / that occurred in this place/ this year/.

The final score of this modified version was the mean of the scores in each story. Only the immediate recall of the stories was requested.

“Na madrugada/ de quinta-feira/, um ônibus/ que ia do Rio/ a São Paulo/, bateu/ em um caminhão/ carregado de cimento/. Treze passageiros/ ficaram feridos/, sendo dois/ em estado grave/. O motorista do caminhão/ faleceu/ no local/. Segundo o comandante da polícia rodoviária/, o ônibus/ estava com velocidade excessiva/ e havia neblina na estrada/. Este é o terceiro acidente/ de graves proporções/ que ocorre nesse local/ neste ano/.”

### Statistical analysis

The Mann-Whitney test was used to compare the scores on the neuropsychological tests according to educational level, and for the M-LMI also according to gender. Spearman’s correlation test was employed for correlating the scores in the M-LMI with age and education. Logistic regression was applied to investigate the influence of age and education on the performance on the M-LMI. The value of significance accepted was 0.05. The software package SPSS for Windows 14.0 was used for the statistical analysis.

## Results

Table 1 shows the average demographic data and scores on the neuropsychological tests of the 363 participants (214 women; 149 men).

**Table 1:** Demographic data and scores on the neuropsychological tests of the 363 participants.

	Mean (SD)	Median	Min-Max	25^th^-75^th^ percentiles
Age	49.2 (13.9)	50	16-87	42-58
Schooling years	6.8 (4.8)	6	0-20	3-10
Mini-Mental State Examination	28.0 (2.6)	29	17-30	27-30
Verbal Fluency (animals)	19.7 (7.3)	19	6-44	15-23
Trail-making-A (time; s)	82.4 (55.9)	61	15-300*	45-105
Fist-palm-edge test^†^	2.1 (1.1)	2	1-5^‡^	1-3
Modified Logical Memory I	9.4 (4.6)	8.5	1-23	5.5-12

Min-Max, minimum and maximum values; MMSE: Mini-Mental State Examination; SD, standard deviation;

* Test was interrupted at 300 s;

† Score was the number of demonstrations needed to obtain correct performance;

‡ Test was interrupted after five demonstrations.

The scores on the M-LMI did not differ for gender, with medians of 8.5 for women and 9.0 for men (p>0.2).

Table 2 shows the mean and median scores of the neuropsychological tests when the variable “years of schooling” was divided into below the median, and greater than or equal to the median. There were statistically significant differences between the performances of the two groups on all the neuropsychological tests (p<0.001).

**Table 2:** Scores on neuropsychological tests according to years of schooling, divided into below the median (N=205) and greater than or equal to the median (N=158).

	Mean (SD)	Median	Min-Max	25^th^-75^th^ Percentiles
Mini-Mental State Examination				
< median of sch. yrs.	27.2 (2.9)	28	17-30	26-30
≥ median of sch. yrs.	29.1 (1.6)	30	20-30	29-30
Verbal Fluency (animals)				
< median of sch. yrs.	17.4 (5.8)	17	6-42	14-20
≥ median of sch. yrs.	22.6 (8.1)	22	9-44	17-28
Trail-making-A (time; s)				
< median of sch. yrs.	102.1 (59.9)	80.0	25-300*	57.5-135
≥ median of sch. yrs.	56.9 (37.1)	48.5	15-240	35-61.2
Fist-palm-edge test^†^				
< median of sch. yrs.	2.3 (1.1)	2	1-5^‡^	2-3
≥ median of sch. yrs.	1.8 (1.0)	2	1-5	1-2
Modified Logical Memory I				
< median of sch. yrs.	7.6 (3.5)	7.01	1-20.5	5.5-12
≥ median of sch. yrs.	11.6 (4.8)	1.5	2-23	8-15

Differences between the two groups of schooling years were significant across all neuropsychological tests (p<0.001; Mann-Whitney test); Min-Max: minimum and maximum values; SD: standard deviation;

* Test was interrupted at 300 s.;

† Score was the number of demonstrations needed to obtain correct performance;

‡ Test was interrupted after five demonstrations.; sch. yrs., schooling years.

There was an inverse correlation between scores on the M-LMI and age (rho= –0.184; p<0.001) and direct correlation between scores on the M-LMI and years of schooling attained (rho=0.524; p<0.001).

Because there was an inverse correlation between age and years of schooling attained in this sample (rho= –0.368; p<0.001), logistic regression was used with the scores on the M-LMI being divided into below the median, and greater than or equal to the median. Age and gender were not included in the equation while only years of schooling attained was included (B=0.173, standard error=0.27; Exponential (B)=1.189, 95% CI 1.128–1253; p<0.001).

There were 23 (15 women) illiterate subjects (23/363) in this sample, with mean age of 59.4 (±7.4) years and median of 60 years. Illiterates were older than the other (literate) participants, whose mean age was 48.5 years (±14.0) and median was 49 years (p<0.001). The performance of the illiterates on the M-LMI was very low with a mean of 5.7 (±2.2) and median of 5.5, in contrast to the other participants who scored 9.6 (±4.6) with median of 9 (p<0.001).

## Discussion

The results showed a major influence of educational level on performance in immediate recall of the stories. The influence of illiteracy was marked.

Other authors have previously reported that education has a greater effect than age on the retention of items in logical memory, both for form 1 and form II,^14-16^ but other authors have not observed any influence of education. 17 The absence of effect of educational level reported in the cited study was probably related to relatively higher range of years of schooling (6–20 years), with a mean of 13.5 years (SD=2.2),^17^ versus this and other studies.^14,16^

In populations with heterogeneous educational background, a very frequent occurrence in developing countries, the evaluation of memory with the recall of short stories is not recommended. Alternatively, if these tests are to be used, scores adjusted for education should be employed.

Although this study did not compare the performance on the M-LMI with that of other memory tests, it is probable that in tests with larger encoding phases, such as the memory tests of the CERAD battery,^18^ and the Brief Cognitive Battery,^19,20^ the Free and Cued Selective Reminding Test^21^ and the Double Memory Test,^22^ are more appropriate for the evaluation of memory impairment in populations with heterogeneous educational level. In the CERAD battery and the Brief Cognitive Battery, the items to be reminded are presented three times to improve encoding,^18,20^ whereas in the Free and Cued Selective Reminding Test as well as in the Double Memory Test semantic encoding is stimulated.^21,22^

The reasons for the poor performance of illiterate and low educated individuals on the immediate recall of short stories is not clear. It is possible that their attention had been focusing on the general meaning of the stories rather than trying to segment the information to encode it. An alternative explanation is that there were difficulties on retelling the stories, which is mainly an executive function. As we did not evaluate the recognition of the items or the recall of the meaning of the stories it is not possible to ascertain the relative contributions of these factors. The artificial setting of the evaluation may have contributed to the poor performance of the low educated participants, especially of the illiterates, but in a previous study we found that the performance of illiterates was no different to that of literates when the memory test of the Brief Cognitive Battery was used.^23^

A limitation of this study was the absence of form II, or delayed recall of the stories, which is more significant for the diagnosis and follow-up of patients with aMCI.^10^ However, the low performance on immediate recall of the M-LMI makes highly probable that the performance on the delayed recall of the stories would also be very poor.

In addition, and from a broader perspective, it is important to consider that much information broadcast by radio or TV, as well as information communicated orally in settings such as hospitals, bus or train stations and airports may be less well retained by low educated individuals, especially when the information is presented only once.

This study was conducted according to the principles established in the Helsinki declaration, but the participants did not sign an informed written consent because this was not a required formal procedure in 1989-1990, when the data were collected.
